# A Third Dose of an Inactivated Vaccine Dramatically Increased the Levels and Decay Times of Anti-SARS-CoV-2 Antibodies, but Disappointingly Declined Again: A Prospective, Longitudinal, Cohort Study at 18 Serial Time Points Over 368 Days

**DOI:** 10.3389/fimmu.2022.876037

**Published:** 2022-04-29

**Authors:** Xian-Ming Liang, Qiu-Yan Xu, Zhi-Juan Jia, Meng-Juan Wu, Yan-Yun Liu, Li-Rong Lin, Li-Li Liu, Tian-Ci Yang

**Affiliations:** ^1^Centre of Clinical Laboratory, Zhongshan Hospital of Xiamen University, School of Medicine, Xiamen University, Xiamen, China; ^2^Institute of Infectious Disease, School of Medicine, Xiamen University, Xiamen, China; ^3^R&D Center, Xiamen Boson Biotech Co., Ltd, Xiamen, China

**Keywords:** COVID-19, SARS-CoV-2, neutralizing antibody, anti-SARS-CoV-2 antibody, CoronaVac

## Abstract

**Background:**

Due to anti-SARS-CoV-2 antibody decay and SARS-CoV-2 variants, vaccine booster doses are a constant concern. It was focused on whether the third dose can quickly evoke and activate immunity and produce a sufficient and durable immune protection.

**Objectives:**

To evaluate the responses and durations of five subsets of anti-SARS-CoV-2 antibodies and their predictive values for protection after the administration of a three-dose inactivated SARS-CoV-2 vaccines regimens.

**Methods:**

A prospective cohort study of five subsets of anti-SARS-CoV-2 antibodies (neutralizing antibody, anti-RBD total antibody, anti-Spike IgG, anti-Spike IgM, and anti-Spike IgA) was carried out to evaluate the efficacies and immune characteristics of a three-dose inactivated SARS-CoV-2 vaccines regimen in 32 volunteers. The dynamic response and immune decay were longitudinally profiled at 18 serial time points over 368 days.

**Results:**

The neutralizing antibody, anti-RBD total antibody, anti-Spike IgG and anti-Spike IgA levels rapidly increased to 773.60 (380.90-1273.00) IU/mL, 639.30 (399.60-878.60) AU/mL, 34.48 (16.83-44.68) S/CO and 0.91 (0.35-1.14) S/CO, respectively, after the administration of the third dose. Compared to the peak value after the second dose, these values were increased by 4.22-fold, 3.71-fold, 1.01-fold and 0.92-fold. On the other hand, the half-lives of the neutralizing antibody, anti-RBD total antibody, and anti-Spike IgG were 56.26 (95% CI, 46.81 to 70.49) days, 66.37 (95% CI, 54.90 to 83.88) days, and 82.91 (95% CI, 63.65 to 118.89) days, respectively. Compared to the half-lives after the second dose, these values were increased by 1.71-fold, 2.00-fold, and 2.93-fold, respectively. Nevertheless, the positive conversion rate of anti-Spike IgM was decreased to 9.38% (3/32), which was much lower than that after the second dose (65.63% (21/32)).

**Conclusions:**

Compared to the second dose, the third dose dramatically increased the antibody levels and decay times. However, the half-life of neutralizing antibody remained unsatisfactory. Due to decay, a fourth dose, and even annual revaccination, might be considered in the SARS-CoV-2 vaccination management strategy.

## Introduction

According to the World Health Organization (WHO), severe acute respiratory syndrome coronavirus 2 (SARS-CoV-2) is estimated to have caused 446.5 million infections and more than 6.0 million deaths over the past 2 years (1). Vaccines are expected to be the most effective and economical means to prevent and control coronavirus disease 2019 (COVID-19) (2). Immunity to SARS-CoV-2 induced through either natural infection or vaccination has been shown to provide some degree of protection against reinfection/infection and reduce the risk of clinical fatality (3). Mass vaccination has played an important role in the effective control of the SARS-CoV-2 epidemic worldwide and protected from infection and severe disease (4–8). The CoronaVac vaccine is an inactivated vaccine and has been approved for emergency use in several countries and has been crucial for curbing the pandemic (9). A recent real-world study conducted in Guangzhou (China) showed that the protection rate of two doses of an inactivated vaccine against delta variant infection exceeded 50% (8).

However, the decline in vaccine efficacy over time remains a major concern (10), as the efficacy of mRNA vaccine was shown to obviously decline gradually over a six-month follow-up period (4). In our previous study, the half-lives of the neutralizing antibody, anti-RBD total antibody, anti-Spike IgG and anti-Spike IgM were 35.61 days, 36.46 days, 30.33 days and 13.54 days, respectively, and the neutralizing antibody seropositive rate dropped to only 19.67% at 160 days after vaccination (11).The decay of vaccine-induced anti-Spike IgG and anti-Spike IgA were faster than that those reported after natural SARS-CoV-2 infection (12). In addition, recently emerged SARS-CoV-2 variants may pose a threat to immunity. Lineage B.1.351 (Beta), P.1 (Gamma) and B.1.617.2 (Delta) significantly escaped natural infection-mediated neutralization, with average live virus neutralization titer reductions of 4.1-fold, 1.8-fold, and 3.2-fold, respectively (13, 14). For B.1.1.529 (Omicron), serum from vaccinees also showed to significant reductions in the geometric mean neutralization titers decreasing from 16.56 to 1.11 after receipt of the second vaccine dose (15). Improving immune responses may be beneficial for combating future SARS-CoV-2 variants (16–18).

Due to the effects of decay and emergence of variants, a three-dose schedule of inactivated vaccine in which a booster dose is administered at least 6 months after the second dose is of constant concern. It is still unknown whether a booster dose of an inactivated vaccine can quickly evoke and activate immunity and produce sufficient and durable immune protection. Here, we conducted a prospective cohort study to longitudinally profile the dynamic responses and decays of five subsets of anti-SARS-CoV-2 antibodies (neutralizing antibody, anti-RBD total antibody, anti-Spike IgG, anti-Spike IgM, and anti-Spike IgA) at 18 serial time points from 32 volunteers over 368 days, and specifically assessed the efficacy and characteristics of the third dose.

## Methods

### Study Design and Participants

We enrolled participants from Xiamen Boson Biotech Co., Ltd., Fujian, China, who were vaccinated with the first standard dose (0.5 mL per dose) of the inactivated CoronaVac vaccine (Sinovac Life Sciences, Beijing, China) on January 24, 2021, vaccinated with the second vaccine dose 28 days later and vaccinated with the third vaccine dose 276 days later (248 days after the second dose). The neutralizing antibody, anti-RBD total antibody (total antibody against the receptor-binding domain (RBD) of the SARS-CoV-2 spike protein), anti-Spike IgG (immunoglobulin G antibody against the spike protein), anti-Spike IgM (immunoglobulin M antibody against the spike protein), and anti-Spike IgA (immunoglobulin A antibody against the spike protein) were serially determined every 7 days for 28 days after every dose and at an additional 5 visits (102 days, 132 days and 248 days after the second dose, 61 days and 92 days after the third dose) to evaluate the immune responses and durations. Sixty-one volunteers received two doses of inactivated vaccination and were initially recruited for a previous study (11). The exclusion criteria included those participants with previous or later SARS-CoV-2 infection, with allergy to any ingredient included in the vaccine, who had received any blood products in the past 4 months, who had received any research medicines or vaccines in the past month, who had uncontrolled epilepsy or other serious neurological diseases, with acute febrile disease, with an acute onset of a chronic diseases, with uncontrolled severe chronic diseases, and who were unable to comply with the study schedule. Finally, only 32 participants received the third vaccine dose and provided blood samples at all 18 serial time points over 368 days.

This study was approved by the Institutional Ethics Committee of Zhongshan Hospital of Xiamen University, School of Medicine, Xiamen University, and was in compliance with national legislation and the Declaration of Helsinki guidelines. All participants provided written informed consent.

### Laboratory Assays

The five subsets of anti-SARS-CoV-2 antibodies were analysed with the reagent matching Autolumo A2000 plus system, which functions based on a chemiluminescence microparticle immunoassay (Anto Biological Pharmacy Enterprise Co., Ltd., Zhengzhou, China) (11). The resulting chemiluminescent reaction was measured as relative light units (RLU). Detection experiments were performed according to the manufacturer’s instructions. The neutralizing antibody assay was based on the one-step competitive method. SARS-CoV-2-specific neutralizing antibodies in the sample bind to an HRP-labeled RBD antigen, which neutralizes the binding of ACE2 (coated on the microparticles) and the RBD antigen. The HRP-labeled RBD antigen not neutralized by SARS-CoV-2-specific neutralizing antibodies forms a complex with ACE2 on the microparticles. The RLU were inversely proportional to the amount of SARS-CoV-2 neutralizing antibody in the sample. The neutralizing antibody level was calibrated and traceable to the First WHO International Standard for anti-SARS-CoV-2 immunoglobulin (NIBSC20/136) and was recorded in international units (IU)/mL (19). Based on 50% protection from SARS-CoV-2 infection, <54.00 IU/mL was considered negative, and ≥54.00 IU/mL was considered positive (3). The anti-RBD total antibody titer was recorded as arbitrary units (AU)/mL based on a 4-parameter fitting method in which the calibration curve was established with the calibrator concentration as the horizontal axis and the calibrator RLU value as the vertical axis, <8.00 AU/mL was considered negative, and ≥8.00 AU/mL was considered positive. The anti-Spike IgG, anti-Spike IgM, and anti-Spike IgA titers were recorded as RLU of the samples/RLU of the cut-off (S/CO), S/CO <1.00 was considered negative, and S/CO ≥1.00 was considered positive.

### Statistical Analysis

Statistical analyses were carried out using IBM SPSS statistics version 26 (SPSS, Inc., Chicago, IL, USA) and GraphPad Prism version 8.00 (GraphPad Software, San Diego, CA, USA). Continuous variables that did not follow a normal distribution are reported as the median with interquartile range (IQR). Repeated-measures ANOVA was conducted to assess the differences in antibody titers over time. The Wilcoxon signed-rank test was used for group comparisons. Fisher’s exact test was used for analysis of the positive conversion rate. The trajectory of antibody titers was fitted by a multilevel model with random intercepts and random slopes. The half-life of antibody titer was assessed over time using the same multilevel modelling approach with R version 3.6.3 (3). A *P* value <0.05 was considered statistically significant.

## Results

### Characteristics of Participants

The clinical data of 32 participants who successfully received the third vaccine dose and provided blood samples at 18 serial time points over 368 days are shown in **Table 1**. The age of the participants ranged from 26 years to 56 years, with a median age of 35 years, and 24 (75.00%) participants were women. All participants were of Han nationality.

**Table 1:** Characteristics of the participants.

Characteristics	Participants (n=32)
Age (years)^a^	35 (31–40)
Sex	
Male	25.00% (8/32)
Female	75.00% (24/32)
Race	
Han nationality	100% (32/32)
Vaccination schedule	
Days between dose 1 and dose 2 (days)	28
Days between dose 2 and dose 3 (days)	248

a Age was recorded as the median with interquartile range (IQR).

### Kinetics of Anti-SARS-CoV-2 Antibodies on a Three-Dose Schedule

**Figure 1:**
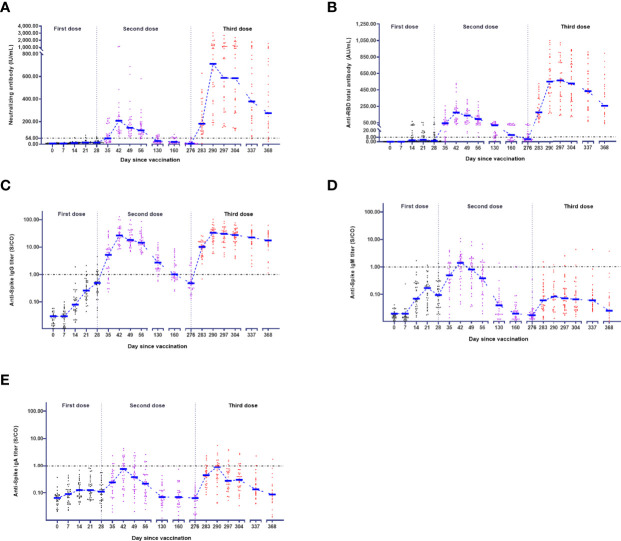
Kinetics of antibodies in the vaccination schedule. The levels of neutralizing antibody **(A)**, anti-RBD total antibody **(B)**, anti-Spike IgG **(C)**, anti-Spike IgM **(D)**, and anti-Spike IgA **(E)** in 32 participants were measured at 18 serial time points over 368 days. The antibody-positive judgement threshold is marked with a line.

The levels of five subsets of anti-SARS-CoV-2 antibodies in 32 participants were measured at 18 serial time points over 368 days. The neutralizing antibody concentration was only 4.75 (2.76 - 11.71) IU/mL with a positive conversion rate of 3.13% (1/32) 248 days after the second dose. After administration of the third dose 248 days after the second dose, the positive conversion rate rapidly reached 96.88% (31/32) in one week. The kinetics of the neutralizing antibody after the third dose was similar to those after the second dose (**Figure 1A**). After both the second and third doses, seroconversion peaked at 100.00% (32/32) (**Table 2**), and the level rapidly increased at two weeks after vaccination, reaching peaks of 207.40 (119.00 - 252.30) IU/mL and 711.90 (380.90 - 1273.00) IU/mL, respectively (**Table 3**). On the other hand, the level also began to decline three weeks after both the second and third doses, declining to the level of 145.50 (103.50-218.10) IU/mL and 587.70 (306.60-1131.00) IU/mL, respectively (**Table 3**).

**Table 2:** Positive seroconversion rates of antibodies according to the vaccination status.

Antibody	First dose	Second dose	Third dose	*P* ^a^	*P* ^b^	*P* ^c^
Neutralizing antibody	9.38% (3/32)	100.00% (32/32)	100.00% (32/32)	0.000	0.000	NA
Anti-RBD total antibody	46.88% (15/32)	100.00% (32/32)	100.00% (32/32)	0.000	0.000	NA
Anti-Spike IgG	21.88% (7/32)	100.00% (32/32)	100.00% (32/32)	0.000	0.000	NA
Anti-Spike IgM	6.25% (2/32)	65.63% (21/32)	9.38% (3/32)	0.000	1.000	0.000
Anti-Spike IgA	0.00% (0/32)	43.75% (14/32)	40.63% (13/32)	0.000	0.000	1.000

a Difference between the first dose and second dose.

b Difference between the first dose and third dose.

c Difference between the second dose and third dose.

NA, not applicable. Fisher’s exact test was used to analyses for the positive conversion rate between the second dose and third dose. However, the test could not be performed due to the number of rows and columns of the crosstab being less than two.

**Table 3:** Anti-SARS-CoV-2 antibodies levels over time after vaccination^a^.

Day since first dose	Day since dose	Neutralizing antibody (IU/mL)	Anti-RBD total antibody (AU/mL)	Anti-Spike IgG (S/CO)	Anti-Spike IgM (S/CO)	Anti-Spike IgA (S/CO)
**First dose**						
D0	D0	6.72 (2.89-9.34)	0.00 (0.00-0.00)	0.03 (0.02-0.03)	0.02 (0.01-0.02)	0.07 (0.04-0.08)
D7	D7	6.72 (3.25-9.84)	0.00 (0.00-0.00)	0.03 (0.01-0.04)	0.02 (0.01-0.02)	0.09 (0.05-0.13)
D14	D14	11.42 (7.37-17.20)	2.51 (0.36-18.67)	0.08 (0.05-0.14)	0.07 (0.03-0.26)	0.13 (0.07-0.23)
D21	D21	14.99 (11.64-19.38)	3.46 (1.18-16.04)	0.26 (0.14-0.59)	0.17 (0.04-0.27)	0.13 (0.07-0.28)
D28	D28	15.04 (10.83-22.28)	1.62 (0.16-12.12)	0.49 (0.22-0.98)	0.10 (0.03-0.3)	0.11 (0.05-0.20)
**Second dose**						
D35	D7	52.45 (24.37-95.41)	44.39 (17.76-87.27)	5.23 (0.88-9.27)	0.50 (0.18-1.35)	0.24 (0.09-0.51)
D42	D14	207.40 (119.00-252.30)	174.90 (83.28-238.20)	26.49 (13.69-48.54)	1.41 (0.40-2.38)	0.74 (0.19-1.55)
D49	D21	145.50 (103.50-218.10)	138.80 (59.14-200.40)	18.09 (13.5-39.60)	0.81 (0.22-1.96)	0.38 (0.13-0.95)
D56	D28	122.80 (85.80-180.20)	96.00 (44.27-138.90)	14.52 (9.87-26.93)	0.39 (0.11-1.13)	0.22 (0.08-0.47)
D130	D102	28.84 (15.90-62.98)	20.37 (10.64-36.00)	2.72 (1.56-5.19)	0.04 (0.02-0.06)	0.07 (0.04-0.18)
D160	D132	20.86 (12.25-52.54)	12.13 (3.72-29.64)	1.02 (0.73-3.08)	0.02 (0.01-0.04)	0.07 (0.04-0.11)
D276	D248	4.75 (2.76-11.71)	4.69 (2.63-14.68)	0.48 (0.31-0.97)	0.02 (0.01-0.02)	0.06 (0.02-0.08)
**Third dose**						
D283	D7	181.50 (117.40-339.20)	177.80 (130.20-353.90)	10.21 (4.81-15.52)	0.06 (0.03-0.18)	0.44 (0.23-0.74)
D290	D14	711.90 (380.90-1273.00)	551.60 (384.00-827.20)	33.50 (16.83-44.16)	0.08 (0.03-0.20)	0.88 (0.35-1.14)
D297	D21	587.70 (306.60-1131.00)	563.00 (339.40-795.00)	30.59 (15.38-39.90)	0.70 (0.03-0.16)	0.27 (0.15-0.71)
D304	D28	584.80 (288.60-1180.00)	525.20 (309.10-760.90)	27.42 (15.73-40.56)	0.06 (0.03-0.13)	0.30 (0.16-0.53)
D337	D61	378.40 (214.10-856.10)	433.00 (214.60-646.70)	22.42 (13.08-35.70)	0.06 (0.03-0.09)	0.13 (0.07-0.26)
D368	D92	275.20 (153.30-725.50)	254.50 (145.60-483.70)	17.50 (10.72-31.77)	0.02 (0.01-0.07)	0.09 (0.05-0.18)
*F*		15.184	8.082	9.313	11.451	5.134
*Degrees of freedom*		17	17	17	17	17
*P^b^*		0.000	0.000	0.000	0.000	0.001

a Antibody levels were recorded as the median with the interquartile range (IQR).

b Repeated measures ANOVA was performed to assess the differences.

For the anti-RBD total antibody, the positive conversion rate was 46.88% (15/32) after the first dose and peaked at 100.00% (32/32) after the second dose or third dose (**Table 2**). Regarding the third dose, the anti-RBD total antibody rapidly increased at three weeks after vaccination, peaked at 563.00 (339.40 - 795.00) AU/mL (**Table 3**), and began to decline four weeks after vaccination (**Figure 1B**).

For anti-Spike IgG, the positive conversion rate was 21.88% (7/32) after the first dose and peaked at 100.00% (32/32) after the second or third dose (**Table 2**). Moreover, the dynamics after the second or third dose were similar to those of the neutralizing antibody (**Figure 1C**). However, the dynamics for anti-Spike IgM were much different from those described above (**Figure 1D**). After the third dose, the positive conversion rate of anti-Spike IgM was decreased to 9.38% (3/32), which was much lower than that in the second dose (65.63% (21/32)) (**Table 2**). For anti-Spike IgA, the seroconversion rate was 0.00% (0/32) after the first dose, 43.75% (14/32) after the second dose and 40.63% (13/32) after the third dose (**Table 2**). The dynamics of anti-Spike IgA after the third dose was similar to those after the second dose (**Figure 1E**).

### Enhance of Responses After the Third Dose

**Figure 2:**
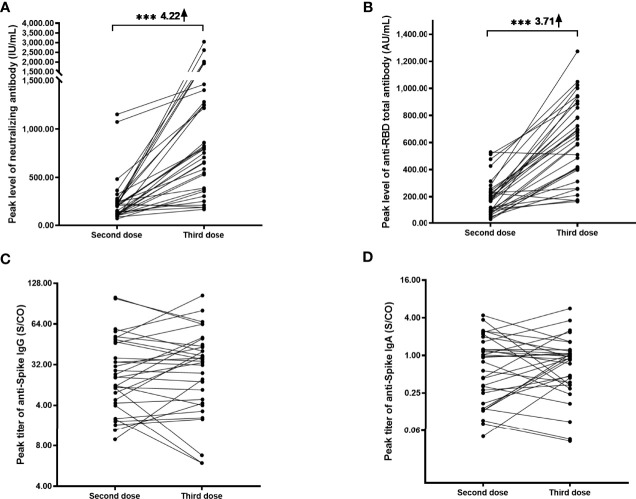
Longitudinal responses of anti-SARS-CoV-2 antibodies after the second and third doses. Paired peak levels of neutralizing antibody **(A)**, anti-RBD total antibody **(B)**, anti-Spike IgG **(C)**, and anti-Spike IgA **(D)** after the second and third doses in a cohort of 32 participants. Because anti-spike IgM showed a minimal response after the third dose, the longitudinal responses failed to be drawn. ***P< 0.001.

To more accurately assess the response, the peak value from the peak level of each participant was more deeply investigated. After the third dose, the neutralizing antibody level rapidly increased from a base value of 4.75 (2.76 - 14.53) IU/mL to a peak value of 773.6 (380.90-1273.00) IU/mL (**Figure 2A**), which was 4.22-fold (95% CI: 2.43-5.31) higher than the peak value (209.70 (119.90-252.40) IU/mL) after the second dose (**Table 4**). For the anti-RBD total antibody, the peak value after the third dose was 639.30 (399.60-878.60) AU/mL, which was 3.71-fold (95% CI: 2.62-4.84) higher than the peak value (174.90 (93.57-238.20) AU/mL) after the second dose (**Figure 2B**). Enhanced neutralizing antibody and anti-RBD total antibody responses were obvious. On the other hand, the responses of the anti-Spike IgG and anti-Spike IgA responses after the third dose were similar to those after the second dose (**Figures 2C, D**). Anti-Spike IgM showed a minimal response after the third dose, and the seropositive rate was only 9.38% (3/32).

**Table 4:** Longitudinal antibodies fluctuation between the second and third doses.

Antibody	Baseline	Peak	Fold change in peak level of the third dose *vs* second dose	*P* ^a^
Neutralizing antibody (IU/mL)				
Second dose	15.04 (10.83-22.28)	209.70 (119.90-252.40)	1.00	
Third dose	4.75 (2.76-14.53)	773.60 (380.90-1273.00)	4.22 (95%CI: 2.43-5.31)	<0.001
Anti-RBD total antibody (AU/mL)				
Second dose	1.62 (0.15-12.12)	174.90 (93.57-238.20)	1.00	
Third dose	4.69 (2.63-14.68)	639.30 (399.60-878.60)	3.71 (95%CI: 2.62-4.84)	<0.001
Anti-Spike IgG (S/CO)				
Second dose	0.49 (0.22-0.98)	26.49 (16.84-48.54)	1.00	
Third dose	0.48 (0.31-0.97)	34.48 (16.83-44.68)	1.01 (95%CI: 0.76-1.38)	0.896
Anti-Spike IgA (S/CO)				
Second dose	0.11 (0.05-0.20)	0.86 (0.19-1.55)	1.00	
Third dose	0.06 (0.02-0.08)	0.91 (0.35-1.14)	0.92 (95%CI: 0.60-2.30)	0.837

a The Wilcoxon signed-rank test was used for group comparisons.

### Decay of Anti-SARS-CoV-2 Antibodies After the Third Dose

**Figure 3:**
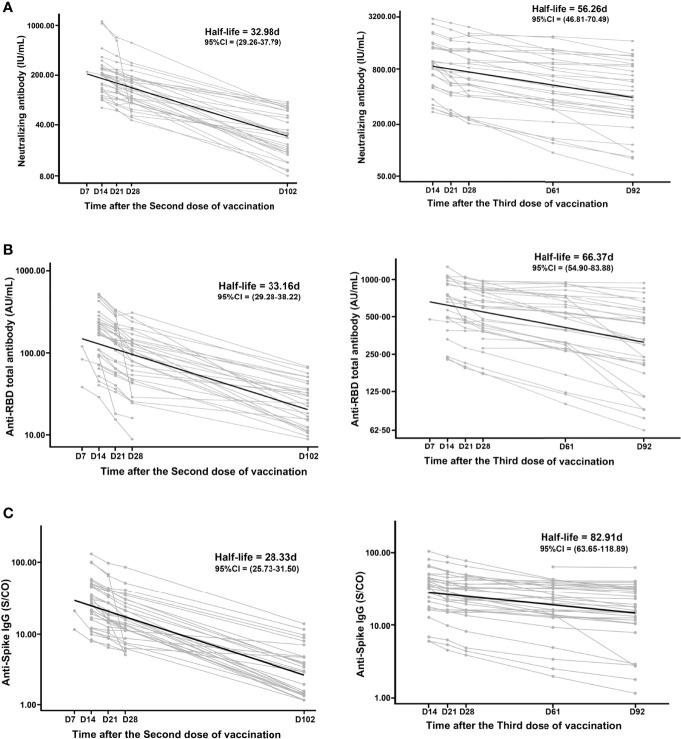
Decay of the anti-SARS-CoV-2 antibodies after the second and third doses. The decay half-lives of neutralizing antibody **(A)**, anti-RBD total antibody **(B)**, and anti-Spike IgG **(C)** were estimated using a linear mixed effects model with censoring of titers above the positive threshold. Due to the small amount of data, the decay half-lives of anti-Spike IgM and anti-Spike IgA failed to fit the model.

To measure anti-SARS-CoV-2 antibody decay after vaccination, we fitted a model of exponential decay and performed half-life analyses based on antibody levels at the first five serial time points after the administration of the second and third doses (**Figure 3**). The neutralizing antibody, anti-RBD total antibody, and anti-Spike IgG half-lives were 56.26 (95% CI, 46.81 to 70.49) days, 66.37 (95% CI, 54.90 to 83.88) days, and 82.91 (95% CI, 63.65 to 118.89) days after the administration of the third dose within 92 days, respectively. These half-lives were longer than those of the neutralizing antibody, anti-RBD total antibody, and anti-Spike IgG after the second dose, which were 32.98 (95% CI, 29.26 to 37.79) days, 33.16 (95% CI, 29.28 to 38.22) days, and 28.33 (95% CI, 25.73 to 31.50) days within 102 days, respectively. These values increased by 1.71-fold, 2.00-fold, and 2.93-fold, respectively. Due to the small amount of data, the fitting curves and decay half-lives of anti-Spike IgM and anti-Spike IgA failed to fit the model, and we were unable to estimate their decay half-lives.

## Discussion

With the emergence of SARS-CoV-2 variants and decay of immunity, vaccine efficacies have weakened, thus requiring a booster dose (20–23). The efficacy of the booster dose is the focus of controlling the COVID-19 epidemic worldwide. In our study, the neutralizing antibody was only 4.75 IU/mL with a serum positivity rate of 3.12% 248 days after the second dose, but a rate of 96.88% was rapidly reached within one week of the third dose. After the third dose, the neutralizing antibody and anti-RBD total antibody levels rapidly increased to 773.60 IU/mL and 639.30 AU/mL, with 4.22-fold and 3.71-fold increases compared to those after the second dose, respectively. On the other hand, decay of the antibody was obvious. The half-lives of the neutralizing antibody, anti-RBD total antibody, and anti-Spike IgG were 56.26 (95% CI, 46.81 to 70.49) days, 66.37 (95% CI, 54.90 to 83.88) days, and 82.91 (95% CI, 63.65 to 118.89) days, respectively. Compared to the half-lives after the second dose, these values increased by 1.71-fold, 2.00-fold, and 2.93-fold. Our results showed that the immune response was more intense and that the decay was slower after the third dose.

Neutralizing antibody levels are highly predictive of immune protection (24). The threshold of the neutralizing antibody level for 50% protection was considered to be 54.00 IU/mL (3). In our study, the neutralizing antibody serum positive conversion rate reached 100% two weeks after the second dose and third doses. After booster vaccination, the majority of individuals reproduced neutralizing antibodies to prevent infection. Due to the limitations of anti-SARS-CoV-2 antibody decay and virus variant emergence, improving immune responses may be beneficial (25–27). The focus was on whether the third dose could quickly evoke the immune memory and increase response. With the BNT162b2 mRNA COVID-19 vaccine, one month after the third dose, neutralization geometric mean titers (GMTs) against wild-type virus increased to more than 7.00-fold higher than the GMTs one month after the second dose (28). In our study, the neutralizing antibody level quickly increased two weeks after the third dose, which was similar to the second dose, suggesting that immune memory was quickly evoked. In addition, the seroconversion rate rapidly reached 100% within two weeks from a baseline of 3.12%. Moreover, the peak level of the neutralizing antibody markedly increased from 209.70 IU/mL after the second dose to 773.60 IU/mL after the third dose, which was a 4.22-fold increase. This result was similar to the report that neutralization GMTs increased from 42.9 on day 28 after the second dose to 158.5 on day 28 following the third dose in inactivated COVID-19 vaccine, which was a 3.69-fold increase (21). A recent real-world study conducted in Guangzhou (China) showed that the protection rate of two doses of an inactivated vaccine against delta variant infection exceeded 50% (8). Given that the level of neutralizing antibody after the third dose was significantly higher than that after the second dose, we believe that the third dose can improve immunity and increase the level of neutralizing antibodies against reinfection/infection.

On the other hand, the duration of immunity after vaccination is vital and used to estimate the protective effects of vaccination. Recent studies have revealed a gradual decrease in the neutralization titer for up to 8 months after SARS-CoV-2 infection (11, 12, 29, 30). In our study, the neutralizing antibody seropositivity rate was only 3.12% 248 days after the second dose, which was lower than that at 132 days (19.67%) in our previous study (11), and the ability to protect against infection worsened. Goel, R. R. reported that recall responses to vaccination in individuals with preexisting immunity primarily increased antibody levels without substantially altering the antibody decay rates (18). To the best of our knowledge, the decay rate of the third dose has not been evaluated. In our study, the half-lives of the neutralizing antibody, anti-RBD total antibody, and anti-Spike IgG were increased 1.71-fold, 2.00-fold and 2.93-fold, respectively, compared with those after the second dose. Our findings supported that the recall responses to boost doses in individuals with preexisting immunity primarily increased antibody levels and substantially altered antibody decay rates. However, in terms of immunity persistence, the half-lives of the antibodies remained unsatisfactory. Due to decay, a fourth dose, or even annual revaccination, might be considered in the SARS-CoV-2 vaccination management strategy.

It is generally accepted that IgM antibodies provide an early-stage response during viral infections prior to the maturation of the class-switched, high affinity IgG response for long-term immunity and immunological memory (31). There are limited data on the kinetics of the appearance of IgM after vaccination and its association with virus neutralizing activity (32). With the BNT162b2 mRNA COVID-19 vaccine, the positive conversion rate for anti-Spike IgM after the second dose was 63.82% (33). In our study, the positive conversion rate after the second dose was 65.63%. Notably, the positive conversion rate was decreased to 9.38% after the third dose. It suggested that response of anti-Spike IgM may be influenced by pre-existing immunity.

To the best of our knowledge, this is the first prospective cohort study of five subsets of anti-SARS-CoV-2 antibodies to evaluate immunity after three-dose vaccination. However, this study has several limitations that need to be addressed. First, only 32 uninfected volunteers were enrolled, thus making the number of participants relatively limited. The results from this study can be applied to this cohort perhaps on cohort where the same vaccination strategy was used, but it cannot be really generalized. Second, immune cells, neutralizing titers against SARS-CoV-2 variants and heterologous vaccination were not investigated in the study. Third, this work lacks a functional correlate, such as antibody-mediated neutralizing activity. Finally, the study span was only 92 days after the third dose.

In conclusion, our results showed that the third dose of vaccine could dramatically increase antibody levels and prolonged the decay time. However, the half-life of neutralizing antibody remained very unsatisfactory. Due to a decline in the immune response, a fourth dose, and even annual revaccination, might be considered in the SARS-CoV-2 vaccination strategy in the future. These findings provide a meaningful basis for the understanding the effects of a booster dose on COVID-19.

## Data Availability Statement

The raw data supporting the conclusions of this article will be made available by the authors, without undue reservation.

## Ethics Statement

The studies involving human participants were reviewed and approved by the Institutional Ethics Committee of Zhongshan Hospital of Xiamen University, School of Medicine, Xiamen University. The patients/participants provided their written informed consent to participate in this study. Written informed consent was obtained from the individual(s) for the publication of any potentially identifiable images or data included in this article.

## Author Contributions

T-CY had full access to all of the data in the study and take responsibility for the integrity of the data and the accuracy of the data analysis. T-CY, L-LL, L-RL, and X-ML supported funding. X-ML, Q-YX, and T-CY contributed to the protocol and design of the study. All authors designed and conducted the statistical analysis and accessed and verified the underlying data. X-ML, Q-YX, and T-CY drafted the manuscript. All authors critically revised the manuscript for important intellectual content. Z-JJ, M-JW, and Y-YL managed participants. All authors contributed to the article and approved the submitted version.

## Funding

This work was supported by the National Natural Science Foundation [grant numbers 81973104, 81772260, and 82003512], the Key Projects for Science and Technology Program of Fujian Province [grant numbers 2021J02055 and 2020J011208], the project for Xiamen Science and Technology Program of Fujian [grant number 3502Z20184057], and the project for Xiamen Medical and Health Guidance [grant number 3502Z20214ZD1037]. The funders had no role in the study design, data collection and analysis, decision to publish, or preparation of the manuscript.

## Conflict of Interest

Authors ZJ-J, MJ-W and Y-YL are employed by Xiamen Boson Biotech Co., Ltd.

The remaining authors declare that the research was conducted in the absence of any commercial or financial relationships that could be construed as a potential conflict of interest.

## Publisher’s Note

All claims expressed in this article are solely those of the authors and do not necessarily represent those of their affiliated organizations, or those of the publisher, the editors and the reviewers. Any product that may be evaluated in this article, or claim that may be made by its manufacturer, is not guaranteed or endorsed by the publisher.
